# International curriculum for undergraduate sonographer education in China during the COVID-19 era: International remote teaching mode vs. domestic on-site teaching mode

**DOI:** 10.3389/fpubh.2022.1083108

**Published:** 2022-12-09

**Authors:** Tingting Qiu, Qiang Lu, Yan Luo, Wenwu Ling

**Affiliations:** Department of Medical Ultrasound, Sichuan University West China Hospital, Chengdu, China

**Keywords:** undergraduate medical students, sonographer education, teaching mode, COVID-19, remote teaching and online learning, on-site teaching

## Abstract

**Background:**

Sichuan University West China Medical School was the first institution in China to develop an undergraduate sonographer education program in 2016. This program was certificated by American Registry for Diagnostic Medical Sonography (ARDMS) and students are qualified for the ARDMS credential verification test. In this 4-year program, the international curriculum of ultrasound physics and hemodynamics was set for students in the third year since 2018. This study is aimed to compare the teaching effect of international remote teaching mode and domestic on-site teaching mode of this international curriculum before and during the COVID-19 pandemic.

**Methods:**

All undergraduate sonographer students after completing ultrasound physics and hemodynamics in the academic years 2018–2019 (30 students; before the COVID-19 pandemic) and 2020–2021 (47 students; during the COVID-19 pandemic) were included in the study. The scores of 77 students were analyzed for their curriculum. Independent samples *t*-test or Mann–Whitney test was employed to compare students' scores before and during the COVID-19 pandemic. The Chi-square test was used to compare students' feedback about this curriculum through an online self-administered questionnaire. A *p* < 0.05 was considered statistically significant.

**Results:**

Total scores were comprised of four parts: in-class tests, homework, mid-term, and final exam scores. The mean in-class test score for domestic on-site teaching mode during the COVID-19 pandemic was significantly higher than that for international remote teaching mode before the COVID-19 pandemic. However, there was no observed a statistically significant difference in homework, mid-term, final exam, and total scores between the two types of teaching modes. For questionnaire feedback, no significant difference was observed between the two groups regarding the satisfaction toward teachers, class atmosphere, teaching mode, curriculum content, exam difficulty, scores, and knowledge students gained. For the overall evaluation of the curriculum, 73.3% (22/30) of students were very satisfied before the COVID-19 pandemic, while 44.7% (21/47) of students felt very satisfied during the COVID-19 pandemic (*p* = 0.02).

**Conclusion:**

The general teaching effect of domestic on-site teaching mode during the COVID-19 pandemic was comparable to that of international remote teaching mode before the COVID-19 pandemic, and domestic on-site teaching mode may provide a better in-class teaching effect.

## Introduction

Coronavirus disease 2019 (COVID-19) was declared a pandemic by the World Health Organization (WHO) since its existence on 11 March 2020, with reported so far over 600 million cases around the world and over 6.5 million deaths (1). During the COVID-19 era, the educational environment has experienced long-lasting changes. Teaching *via* the internet has mostly replaced classroom traditional face-to-face lectures (2–4). Although online distance learning is reported to be acceptable and may increase the retention of information, the absence of a classroom learning atmosphere and a decrease in on-site interaction with teachers or classmates is questionable (5, 6). With the adaptation of mass vaccinations and strict anti-virus actions in China, the COVID-19 pandemic reached a plateau, which provided chances for face-to-face classroom teaching.

Sichuan University West China Medical School was the first medical educational institution in China to develop an undergraduate sonographer education program in 2016. This is a certificate program provided by American Registry for Diagnostic Medical Sonography (ARDMS) for which the students have to qualify for the ARDMS credential verification test. Based on the cooperation with Thomas Jefferson University, this program follows a student-centered, problem-based, and clinical practice-oriented curriculum. International curriculum ultrasound physics and hemodynamics is a foundation and an integral part of the academic courses, which is also a prerequisite for the Sonography Principle and Instrumentation test of ARDMS (7, 8). It is scheduled for the third-year students of this 4-year program. This program enhances the knowledge of the students of ultrasound techniques and their applications in clinical medicine and propels the certification of the role of a sonographer in the Chinese medical system.

As a new education program in China, the basic curriculum of ultrasound physics and hemodynamics was first delivered online *via* cisco Webex by an experienced sonographer educator from Thomas Jefferson University in the autumn semester of 2018 and 2019. However, after the COVID-19 outbreak, this online international remote teaching mode was unsustainable. Therefore, domestic on-site teaching mode was used in 2020 and 2021.

## Materials and methods

### Study design

This retrospective study compared the teaching effect of two types of teaching modes (international remote teaching mode *vs*. domestic on-site teaching mode) for undergraduate sonographer students before and during the COVID-19 pandemic. In-class tests, homework, and mid-term and final exam papers were submitted by these students upon completing the international curriculum ultrasound physics and hemodynamics in the academic years 2018–2019 (before the COVID-19 pandemic) and 2020–2021 (during the COVID-19 pandemic). The scores of these papers were analyzed in order to study the difference, if any, in students' performance toward the different teaching modes for this international curriculum. An online self-administered questionnaire was used to collect feedback from students after they completed this curriculum.

### Curriculum setting

This international curriculum was composed of 8 chapters including physical principles, interactions of sound with tissue, ultrasound transducers, transducer care and maintenance, imaging principles and instrumentation, the transmission of ultrasound, reception of ultrasound, imaging artifact, Doppler imaging concepts, quality assurance and lab accreditation, and new technologies. Each chapter was introduced in a 90-min class each week. The in-class test was a collaborative assignment consisting of 10 single-choice questions carried out for 10–15 min in class. Homework was an independent assignment containing 15 questions (question types: filling in the blanks, true or false, single choice) after class. The mid-term exam was a review of the first 4 chapters' contents and this test was made up of 50 single-choice questions. The final exam paper was designed using single-choice, multiple-choice, short-answer, and subjective questions, which was a summary of this curriculum. All tests were in English. Total scores for each student were composed of in-class test average score (30%), homework average score (30%), mid-term (10%), and final exam score (30%).

### Teaching mode

**Figure 1:**
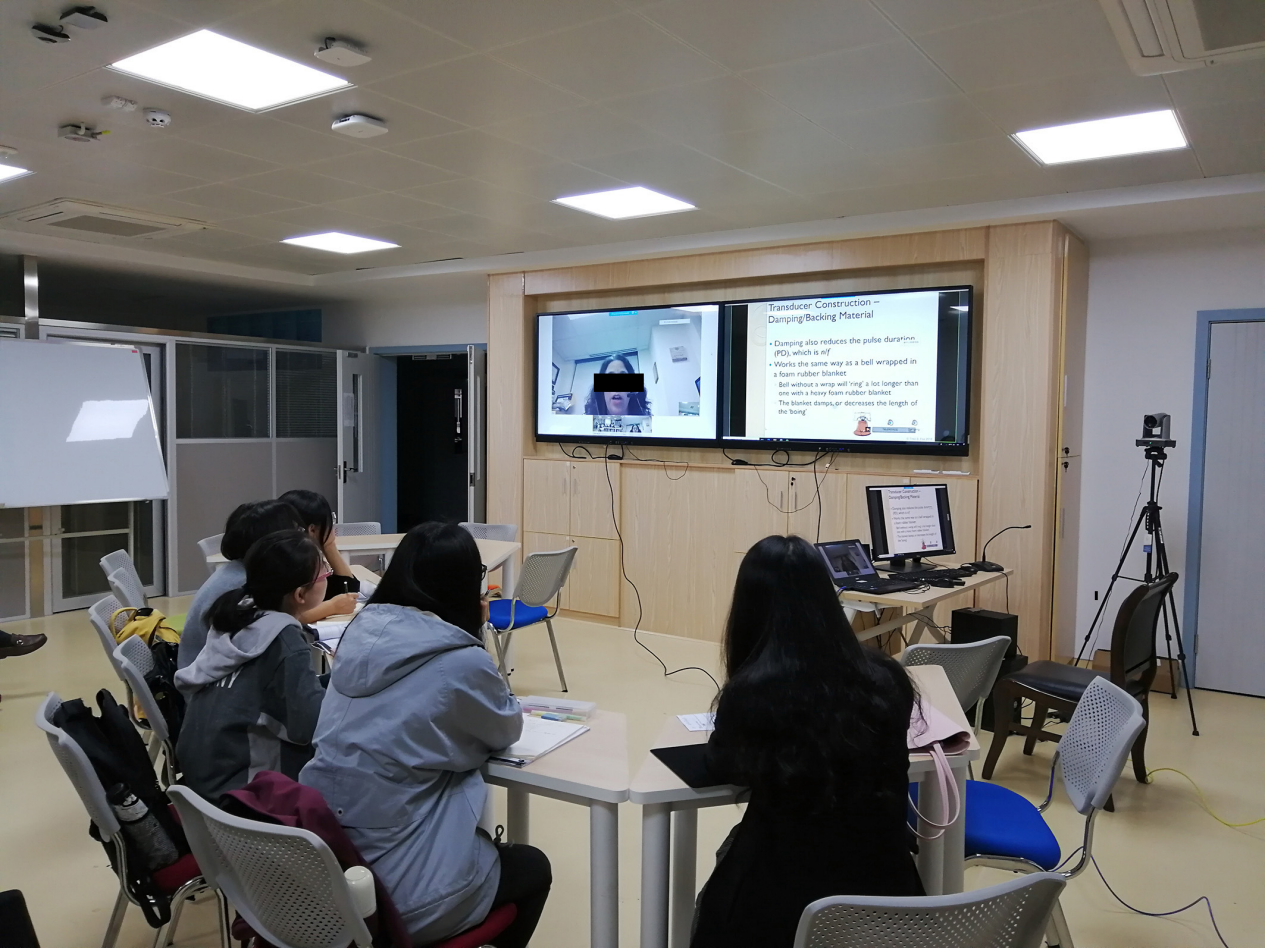
International remote teaching mode, this mode was performed through a live video lecture by an American associate professor completely in English.

**Figure 2:**
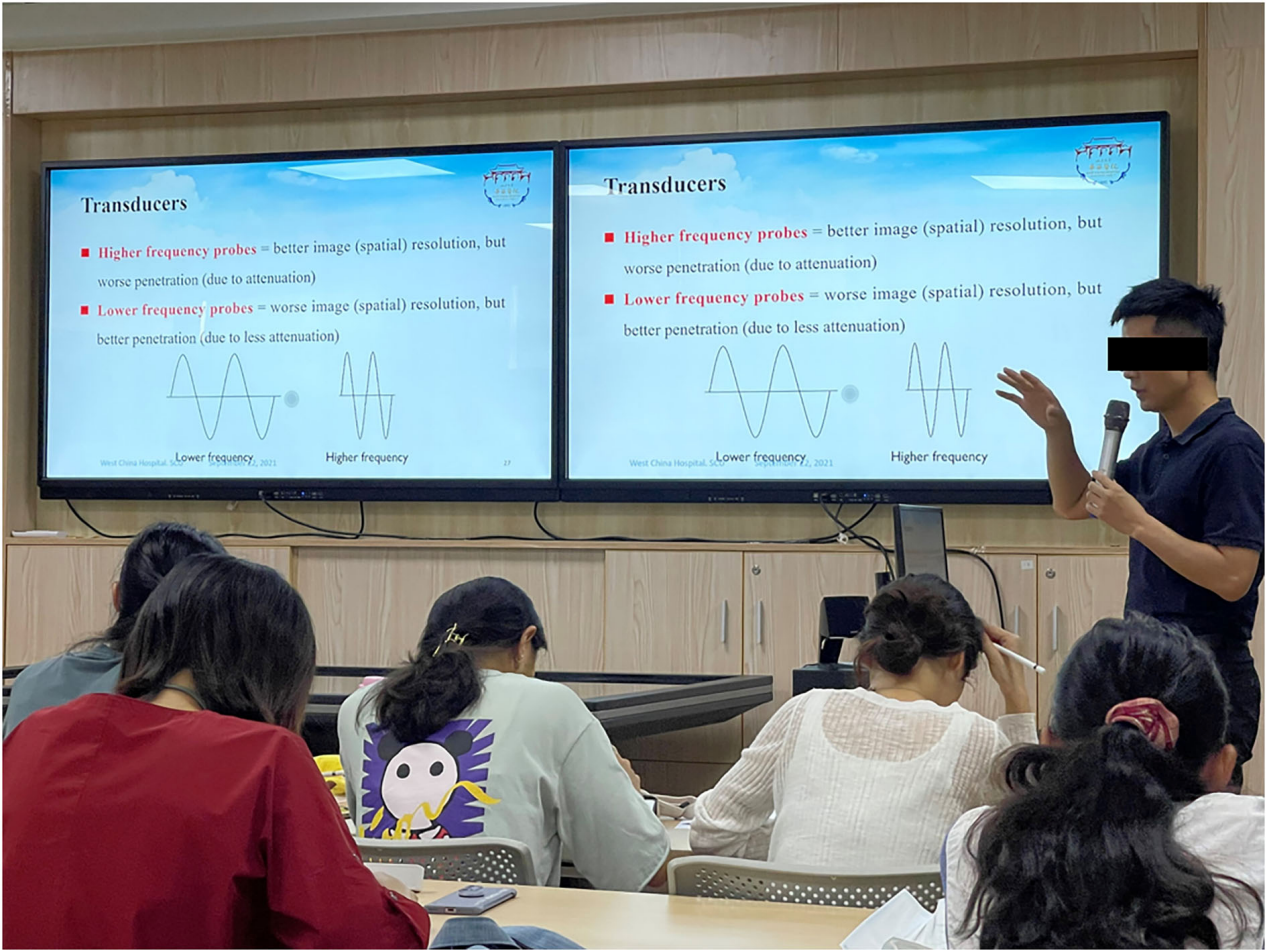
Domestic on-site teaching mode, this mode was carried out by a Chinese teacher in English.

In the academic years 2018–2019 (before the COVID-19 pandemic), international remote teaching mode was used for each class. This mode was performed through a live broadcast lecture by an American associate professor in full English (Figure 1). In the academic years 2020–2021 (during the COVID-19 pandemic), a domestic on-site teaching mode was applied for each class. This on-site teaching mode was carried out by four Chinese teachers (two professors, one associate professor, and one lecturer) in English. Each Chinese teacher was responsible for handling two chapters (Figure 2). All the students and Chinese teachers passed the College English Test Band-6 exam. All the Chinese teachers were found to have fluent oral English.

### Online questionnaire

An online self-administered questionnaire (Supplementary material) was developed to evaluate the students' feedback upon completing the international curriculum for ultrasound physics and hemodynamics. The completion of this questionnaire was not mandatory but strongly encouraged. The questionnaire consisted of two main sections: the first part was intended to collect general information, whereas the second part was designed to collect students' feedback about the curriculum before the COVID-19 pandemic and during the pandemic by using eight items. For detailed information about those items and response options of the questionnaire items, please refer to the Supplementary material.

### Sample size

All undergraduate sonographer students who have completed the international curriculum ultrasound physics and hemodynamics in the academic years 2018–2019 (30 students; before the COVID-19 pandemic) and 2020–2021 (47 students; during the COVID-19 pandemic) were included in the study. The scores and responses of 77 students were analyzed for this curriculum.

### Data collection

Data were collected based on in-class tests, homework, mid-term, and final exam papers along with online self-administered questionnaires received for the academic years 2018–2019 and 2020–2021.

### Statistical analysis

Statistical analysis was conducted using GraphPad Prism, version 8.0. Continuous variables were presented as mean and standard deviation (SD) or median and interquartile range. Categorical variables were presented as frequencies and percentages. Unpaired *t*-test or Mann–Whitney test was used to compare students' performance on international curriculum ultrasound physics and hemodynamics before and during the COVID-19 pandemic. The Chi-square test or Fisher's exact test was used to compare students' responses to the questionnaire items. A *p* < 0.05 was considered statistically significant.

### Ethical considerations

This study was approved by the Research and Ethics Committee of West China Hospital of Sichuan University. The names of students were kept anonymous. All data were kept confidential.

## Results

### Comparison of students' scores for ultrasound physics and hemodynamics before and during the COVID-19 pandemic

A total of 77 undergraduate sonographer students (age range 19–22 years old, female *n* = 55, male *n* = 22) participated in the present study. A total of 30 students participated before the COVID-19 pandemic (international remote teaching mode), and 47 students participated during the COVID-19 pandemic (on-site teaching mode). The average score of the in-class test, homework, mid-term and final exam between the two groups was 93 vs. 97 (*p* = 0.0025), 97 vs. 96 (*p* = 0.3359), 92 vs. 90 (*p* = 0.0943), 74 vs. 76 (*p* = 0.6268). The total score between the two groups was 88 vs. 90 (*p* = 0.2929) (Table 1).

**Table 1:** Test scores of curriculum ultrasound physics and hemodynamics between two teaching modes.

**Teaching mode/test scores (100 points)**		**International remote teaching mode**	**On-site teaching mode**	***p* value**
		**Before COVID-19 pandemic;**	**During COVID-19 pandemic;**	
		***n* = 30**	***n* = 47**	
In-class test	Minimum	81	67	0.0025
	Maximum	98	100	
	Average	93	97	
	Standard deviation	4.1	5.6	
Homework	Minimum	83	86	0.3359
	Maximum	100	99	
	Average	95	95	
	Standard deviation	4.7	3	
Mid-term exam	Minimum	60	78	0.0943
	Maximum	100	100	
	Average	92	90	
	Standard deviation	11.1	7.4	
Final exam	Minimum	22	27	0.6268
	Maximum	98	100	
	Average	74	76	
	Standard deviation	19.6	15.6	
Total score	Minimum	70	70	0.2929
	Maximum	99	98	
	Average	88	90	
	Standard deviation	7.6	5.7	

### Comparison of students' responses toward ultrasound physics and hemodynamics before and during the COVID-19 pandemic

A total of 77 feedbacks for the curriculum were received, of which 30 responses were assessments of the international remote teaching mode before the COVID-19 pandemic. The rest 47 responses reflected students' subjective evaluations of the on-site teaching mode during the COVID-19 pandemic. As illustrated in Table 2, no significant difference was observed between the two groups for the satisfaction toward teachers, class atmosphere, teaching mode, curriculum content, exam difficulty, scores, and knowledge students gained. However, for the overall evaluation of the curriculum, 73.3% (22/30) students were very satisfied before the COVID-19 pandemic, while 44.7% (21/47) students felt very satisfied during the COVID-19 pandemic (*p* = 0.02).

**Table 2:** Satisfaction questionnaire of curriculum ultrasound physics and hemodynamics between two teaching modes.

**Teaching mode/questionnaire**		**International remote teaching mode**		**On-site teaching mode**		***p* value**
		**Before COVID-19 pandemic;**		**During COVID-19 pandemic;**		
		***n*** = **30**		***n*** = **47**		
		***n***	**%**	***n***	**%**	
Curriculum teacher	Very satisfied	23	76.7	29	61.7	0.3133
	Satisfied	7	23.3	16	34	
	Neutral	0	0	1	2.1	
	Not satisfied	0	0	1	2.1	
Class atmosphere	Interesting	24	80	39	83	0.7565
	Neutral	6	20	7	14.9	
	Boring	0	0	1	2.1	
Teaching mode	Very satisfied	19	63.3	24	51.1	0.2076
	Satisfied	7	23.3	18	38.3	
	Neutral	4	13.3	4	8.5	
	Not satisfied	0	0	1	2.1	
Curriculum content difficulty	Difficult	3	10	8	17	0.513
	Neutral	27	90	39	83	
	Easy	0	0	0	0	
Exam difficulty	Difficult	0	0	4	8.5	0.25
	Neutral	29	96.7	41	87.2	
	Easy	1	3.3	2	4.3	
Curriculum scores	Very satisfied	7	23.3	13	27.7	0.8407
	Satisfied	17	56.7	23	48.9	
	Neutral	6	20	10	21.3	
	Not satisfied	0	0	1	2.1	
Curriculum knowledge	Very satisfied	17	56.7	21	44.7	0.3492
	Satisfied	12	40	24	51.1	
	Neutral	1	3.3	2	4.3	
	Not satisfied	0	0	0	0	
General assessment	Very satisfied	22	73.3	21	44.7	0.02
	Satisfied	8	26.7	25	53.2	
	Neutral	0	0	1	2.1	
	Not satisfied	0	0	0	0	

## Discussion

The undergraduate sonographer education program was initiated in China in 2016. Based on the cooperation between West China medical school and Thomas Jefferson University, this program enhanced the sonographer training system and alleviated the shortage of medical ultrasound professionals in China (9–12). In 2018 and 2019, ultrasound physics and hemodynamics, one of the academic courses for this program, was delivered in international remote teaching mode. With the COVID-19 pandemic outbreak, the educational environment has changed (13–15)and domestic on-site face-to-face teaching mode was adopted for this curriculum in the post-COVID-19 era.

This retrospective study compared international remote teaching mode and domestic on-site teaching mode for the theoretical medical curriculum ultrasound physics and hemodynamics. The results of students' scores showed that no significant difference was observed for homework, mid-term, final exam score, and total score between the two types of teaching modes, which indicated that the general teaching effect of the two types of teaching modes was comparable. These results are in line with studies finding that digital transformation of the theoretical medical curriculum can be feasible during the COVID-19 pandemic (16, 17). However, it's noted that students' in-class test score under domestic on-site teaching mode was significantly higher than that under international remote teaching mode. This phenomenon may be due to the more active class learning atmosphere or interactions of on-site face-to-face teaching mode compared to distance learning online (17, 18). Several studies also emphasized the importance of classroom teaching interactions in the teaching process (19–22). Another interesting result was that the average score of the final exam was significantly lower than that of other forms of testing in both international remote teaching mode and domestic on-site teaching mode. Because, first, in-class tests and homework included testing contents of only one chapter, mid-term exams contained testing contents of the first four chapters. The final exam tested the whole eight chapters' key points, which was relatively more difficult. Second, as described in the methods curriculum setting part, the in-class test, homework, and mid-term exam mainly (>90%) contained single-choice question type, while the final exam paper was designed using single-choice, multiple-choice, short-answer, and subjective questions, which also increased difficulty.

Online questionnaire responses revealed that students' stratifications toward teachers, class atmosphere, teaching mode, curriculum content, exam difficulty, scores, and knowledge were similar between the two groups. This indicated that two types of teaching modes were acceptable for this theoretical medical curriculum. However, students before the COVID-19 pandemic expressed significantly higher levels of general satisfaction with the curriculum as compared to that during the COVID-19 pandemic. One possible explanation for this finding is the freshness of this brand-new curriculum and teaching mode in China before the COVID-19 pandemic. Before the COVID-19 outbreak, classroom face-to-face lectures are a daily teaching mode for undergraduate students. Therefore, the international remote teaching mode at that time was unique and interesting for most students. This may lead to higher levels of general satisfaction with the curriculum. Similar feedback was reported by studies on transforming didactic face-to-face lectures into online sessions, workshops, or seminars that appear to be more attractive to medical students (23).

Despite the relatively higher general satisfaction rate among undergraduate sonographer students toward the curriculum under international remote teaching mode in this study, higher in-class test scores under domestic on-site teaching mode highlighted the importance of face-to-face experiences and classroom interactions. As a theoretical medical curriculum, the transformation from international remote teaching mode to domestic on-site teaching mode was successful, which also improved the in-class teaching effect. In the background of the COVID-19 pandemic and within the context of learning medical knowledge, a hybrid of the two types of teaching mode along with hands-on training would be more suitable and considerable in the future. In line with the recommendations from other studies (17, 24), we encourage educators to use hybrid strategies to improve the experiences of medical students in learning medical concepts and skills.

In conclusion, the general teaching effect of domestic on-site teaching mode during the COVID-19 pandemic was comparable to that of international remote teaching mode before the COVID-19 pandemic, and domestic on-site teaching mode may provide better in-class teaching effects.

## Data availability statement

The raw data supporting the conclusions of this article will be made available by the authors, without undue reservation.

## Ethics statement

The studies involving human participants were reviewed and approved by Research and Ethics Committee of West China Hospital of Sichuan University. Written informed consent for participation was not required for this study in accordance with the national legislation and the institutional requirements.

## Author contributions

Conceptualization, funding acquisition, and writing—original draft: TQ. Methodology and writing—review and editing: TQ, QL, YL, and WL. Investigation and project administration: TQ and WL. Visualization: QL, YL, and WL. Supervision: YL and WL. All authors have reviewed and approved the manuscript, contributed to the article, and approved the submitted version.

## Supplementary material

Supplementary Data Sheet 1 Satisfaction questionnaire of international curriculum ultrasound physics and hemodynamics.
