# Supplementary material for: International curriculum for undergraduate sonographer education in China during the COVID-19 era: International remote teaching mode vs. domestic on-site teaching mode
Source: Front Public Health. 2022 Dec 9;10:1083108. doi: 10.3389/fpubh.2022.1083108 (PMC9780291; doi:10.3389/fpubh.2022.1083108)
Supplement: Supplementary Data Sheet 1 — Satisfaction questionnaire of international curriculum ultrasound physics and hemodynamics. [file Data_Sheet_1.docx]

Supplementary Material

Questionnaire

1. In which year you took ultrasound physics and hemodynamics (2018, 2019, 2020 or 2021)?

2. How old were you when you were learning this curriculum?

3. What’s your gender (boy or girl)?

4. Are your satisfied with the teacher for this curriculum?

A. Very satisfied

B. Satisfied

C. Neutral

D. Not satisfied

5. How do you like the class atmosphere?

A. Interesting

B. Neutral

C. Boring

6. Are your satisfied with the teaching mode?

A. Very satisfied

B. Satisfied

C. Neutral

D. Not satisfied

7. How do you like the curriculum content difficulty?

A. Difficult

B. Neutral

C. Easy

8. How do you like the exam difficulty?

A. Difficult

B. Neutral

C. Easy

9. Are your satisfied with the curriculum scores you got?

A. Very satisfied

B. Satisfied

C. Neutral

D. Not satisfied

10. Are your satisfied with the curriculum knowledge you got?

A. Very satisfied

B. Satisfied

C. Neutral

D. Not satisfied

11. What’s your general assessment for this curriculum?

A. Very satisfied

B. Satisfied

C. Neutral

D. Not satisfied
